# Prevalence and determinants of susceptibility to cigarette smoking among school students in Pakistan: secondary analysis of Global Youth Tobacco Survey

**DOI:** 10.1186/1747-597X-9-10

**Published:** 2014-02-21

**Authors:** Syeda Kanwal Aslam, Sidra Zaheer, Saadiyah Rao, Kashif Shafique

**Affiliations:** 1 School of Public Health, Dow University of Health Sciences, OJHA Campus, SUPARCO road, Gulzar e Hijri, Karachi, Pakistan; 2 Institute of Health and Wellbeing, Public Health, University of Glasgow, 1-Lilybank Gardens, Glasgow, G12 8RZ, UK

**Keywords:** Smoking, Adolescents, Students, Susceptibility to cigarette smoking, Pakistan

## Abstract

**Background:**

Susceptibility to smoke has been recognized as a strong predictor of smoking experimentation and taking up regular smoking habit. The identification of smoking susceptible individuals and its determinants is important in the efforts to reduce future smoking prevalence. The aims of this study are to estimate prevalence of susceptibility to smoke among adolescents, and identify factors associated with it.

**Methods:**

Cross sectional data was obtained from Global Youth Tobacco Survey conducted in three cities of Pakistan in year 2004. Study population consisted of students in grades, 8^th^, 9^th^, and 10^th^; aged 13 to 15 years. Secondary analysis using univariate and multivariate logistic regression analyses were performed to estimate the associations between smoking susceptibility and co-variates. Descriptive statistics were reported in proportions, and adjusted odds ratios with 95% confidence interval were used to report logistic regression analyses.

**Results:**

Approximately 12% of nonsmoking students were found susceptible to smoking. Students, who were females (OR = 1.53, 95% CI [1.24-1.89]); whose parents (OR = 1.64, 95% CI [1.35-1.99]); or close friend smoked (OR = 2.77, 95% CI [2.27- 3.40]) were more susceptible to cigarette smoking. Students who had good knowledge about harmful effects of smoking (OR = 0.54, 95% CI [0.43-0.69]); and had access to anti-smoking media (OR = 0.73, 95% CI [0.59-0.89]) were less likely to be susceptible to smoking.

**Conclusion:**

Students who were females, had smoking parents, friends or exposure to newspaper/magazines cigarette marketing, were more susceptible to cigarette smoking among Pakistani adolescents. While knowledge of harmful effects of smoking and access to anti-smoking media served as protective factors against susceptibility to smoking.

## Background

Cigarette smoking is considered as the leading preventable cause of non-communicable diseases and its associated mortality worldwide. Although cigarette smoking has declined globally particularly in the developed countries [1] but most low income countries including Pakistan continue to face increasing burden of tobacco epidemic with a current cigarette smoking prevalence of 15.2% among adults and 6.3% among youth [2,3]. It has been projected that if current trends of smoking prevalence continue, annual tobacco related deaths will be over 8 million by the year 2030, and more than two third of these will occur in low and middle income countries [4].

Majority of the cigarette smokers (88%) start smoking in their teenage, and thus adolescents and young adults remain the focus of preventive efforts [5]. Cigarette smoking is a learned behavior which passes through various stages namely: “preparation, initiation, experimentation, regular smoking, and finally addiction” [6]. Susceptibility to smoke is defined as “lack of firm decision against smoking and usually starts in the preparation and/or initiation stages of smoking behavior”; and has been validated as an important predictor of cigarette experimentation [7] Adolescents who are susceptible to smoke have double the risk of taking up smoking as compared to non-susceptible individuals [8]. Although various social and behavioral factors have been identified as important risk factors of smoking; their importance as predictors of initiation stage of smoking is not well understood in low income countries [9]. Furthermore, limited information is available on susceptibility to smoke and its associated risk factors from low income countries [10,11]. The current scenario calls for in depth understanding of the initial stages of smoking, as youth going through these stages of smoking behavior are at considerable risk of regular cigarette addiction in future. Interestingly, these individuals are in an age group which has great potential to be affected by the primary preventive measures of anti-tobacco programs and policies. Thus, identification of smoking susceptible individuals and its determinants is important in the efforts to reduce future smoking prevalence. Therefore, the aim of this study is to estimate prevalence of susceptibility to smoke among Pakistani school children and identify factors associated with it.

## Methods

GYTS is a school based survey developed by the World Health Organization (WHO) and Centers for Disease Control and Prevention (CDC). We performed secondary analysis on GYTS Pakistan 2004 data. This survey was conducted in three cities of Pakistan, namely Kasur, Peshawar, and Quetta using multistage sample design. At the first stage, the schools were selected proportional to student enrollment size in schools. At the second stage, classrooms were chosen randomly from the schools selected at first stage [12]. Students aged 13 to 15 years are focused in GYTS, and country research coordinators identified the grades, 8^th^, 9^th^, and 10^th^ as the ones that correspond to these ages. CDC and WHO ensured that participating countries follow local procedures for obtaining parental permission for ethical consideration [13]. Data were collected using a self-administered questionnaire, which was developed by CDC and WHO. According to CDC and WHO, sample size has been calculated using the information that a minimum of 1500 completed student interviews are needed to obtain a precision level of ± 5% for a given estimate. This information is then used by the country to determine sample size of schools and students [13]. Detailed information of GYTS Pakistan 2004 is available on the CDC website [12].

The study questions which were selected for analysis included information on age, sex, current smoking status, peer and parents’ smoking status, attitudes and behaviors, information on the knowledge of smoking related health risks, exposure to anti-smoking media messages, exposure to cigarette advertisements, and experimentation with cigarettes. Participants were aged between 11 and 17, and were stratified in two groups of less than or equal to 13, and greater than 13 years. According to the usual school grade system in our country, students up to 13 years of age are in lower secondary school and those older than 13 years are in higher secondary school. Our aim was to study the pattern of susceptibility in these two different school going groups. We measured the outcome variable, susceptibility to smoking using three questions from the survey; a) “If one of your friends offered you a cigarette, would you smoke it?” b) “At any time during the next twelve months, do you think you will smoke a cigarette?” c) “Do you think you will be smoking cigarettes 5 years from now?” Students who are currently non-smokers and answered definitely not to all 3 questions were coded as non-susceptible, and all other students were labeled as susceptible to smoking. All questions and criteria for determining susceptibility to smoking have been adopted from Pierce et al. [7]. Details of the coding plan of all variables used in our study are mentioned in Table 1.

**Table 1:** Coding plan for the selected study variables

**Variable**	**Question in codebook**	**Response options**	**Response options**
**Outcome variable**			
**Susceptibility to smoking**	1. If one of your friends offered you a cigarette, would you smoke it?	1 = Definitely not	Students who are currently non-smokers and answered “Definitely not” to all 3 questions were coded as “non-susceptible”, and all other students were labeled as “susceptible” to smoking.
		2 = Probably not	
		3 = Probably yes	
		3 = Probably yes	
	2. At any time during the next 12 months, do you think you will smoke a cigarette?	2. At any time during the next 12 months, do you think you will smoke a cigarette?	
		2. At any time during the next 12 months, do you think you will smoke a cigarette?	
		3 = Probably yes	
		4 = Definitely yes	
	3. Do you think, you will be smoking a cigarette 5 years now?	1 = Definitely not	
		2 = Probably not	
		3 = Probably yes	
		4 = Definitely yes	
**Independent variables**			
**Age in years (categorical)**	How old are you?	1 = 11 years old or younger	1 = Aged ≤ 13
		2 = 12 years old	2 = Aged > 13
		3 = 13 years old	
		4 = 14 years old	
		5 = 15 years old	
		6 = 16 years old	
		7 = 17 years old or older	
**Parents’ smoking status**	Do your parents smoke?	1 = None	1 = None
		2 = Both	2 = At least one parent smokes
		3 = Father only	3 = I don’t know
		4 = Mother only	
		5 = I don’t know	
**Friends’ smoking status**	Do any of your closest friends smoke cigarettes?	1 = None of them	1 = Do not have friends who smoke
		2 = Some of them	2 = Have friends who smoke
		3 = Most of them	
		4 = All of them	
**Classes on harmful effects of smoking**	During this school year were you taught in any of your classes about the dangers of smoking?	1 = Yes	1 = Did attend
		2 = No	2 = Did not attend
		3 = Not sure	
**Access to anti smoking media**	During the past 30 days (1 month) how many anti-smoking media messages (e.g. television, radio, billboards, posters, newspapers, magazines, movies) have you seen?	1 = A lot	1 = Yes
		2 = A few	2 = No
		3 = None	
**Exposure to billboards cigarette marketing**	During the past 30 days (1 month) how many advertisements for cigarettes have you seen on billboards?	1 = A lot	1 = Yes
		2 = A few	2 = No
		3 = None	
**Exposure to newspapers/magazines cigarette marketing**	During the past 30 days (1 month) how many advertisements or promotions for cigarettes have you seen in newspapers or magazines?	1 = A lot	1 = Yes
		2 = A few	2 = No
		3 = None	
**Boys who smoke have “more or less friends**	Do you think boys who smoke cigarettes have more or less friends?	1 = More friends	1 = More friends
		2 = Less friends	2 = Less friends or no difference
		3 = No difference from nonsmokers	
**Girls who smoke have more or less friends**	Do you think girls who smoke cigarettes have more or less friends?	1 = More friends	1 = More friends
		2 = Less friends	2 = Less friends or no difference
		3 = No difference from nonsmokers	
**Boys who smoke are more or less attractive**	Do you think smoking cigarettes make boys more or less attractive?	1 = More attractive	1 = More attractive
		2 = Less attractive	2 = Less attractive or no difference
		3 = No difference from nonsmokers	
**Girls who smoke are more or less attractive**	Do you think smoking cigarettes make girls more or less attractive?	1 = More attractive	1 = More attractive
		2 = Less attractive	2 = Less attractive or no difference
		3 = No difference from nonsmokers	
**People who smoke are more or less comfortable at social gatherings**	Does smoking cigarettes help people feel more comfortable at celebrations, parties or in other social gatherings?	1 = More comfortable	1 = More comfortable
		2 = Less comfortable	2 = Less comfortable or no difference
		3 = No difference from nonsmokers	
**Knowledge of harmful effects of smoking**	Do you think cigarette smoking is harmful to your health?	1 = Definitely not	0 = Poor knowledge (response 1-3)
		2 = Probably not	1 = Good knowledge (response 4)
		3 = Probably yes	
		4 = Definitely yes	
**Knowledge of harmful effects of secondhand smoke**	Do you think smoke from other people’s cigarette is harmful to you?	1 = Definitely not	0 = Poor knowledge (response 1-3)
		2 = Probably not	1 = Good knowledge (response 4)
		3 = Probably yes	
		4 = Definitely yes	
**Ever tried cigarette smoking**	Have you ever tried or experimented with cigarette smoking, even one or two puffs?	1 = Yes	1 = Ever smoked
		2 = No	0 = Never smoked

### Data analysis

Descriptive and Pearson chi-square analyses were used to determine the associations between smoking susceptibility (dependent variable ) and other independent variables, including student’s demographics, parents’ and friends” influences, education and knowledge of harmful effects of smoking, exposure to antismoking media and advertisements and expected social outcomes of smoking.

In order to identify the variables that predicted susceptibility, we performed univariate and multivariate logistic regression analysis to estimate the significant associations between smoking susceptibility and co-variates. Furthermore, in order to compare the susceptibility among ever smoked and never smoked groups, we performed the multivariate logistic regression with all risk factors that demonstrated a significant association with susceptibility i.e. sex, age, parents’ and friends’ smoking status, knowledge of harmful effects of smoking, access to anti-smoking media, and exposure to cigarette advertisements. Data were analyzed by using SPSS version 16.0.

## Results

Overall, 6204 students participated in GYTS 2004 from Peshawar (2159), Quetta (1804) and Kasur (2241). The school response rate was 100.0%, class response rate was 100.0%, and student response rate was 88.6% on average in all three cities [14]. We constructed our final dataset using information from 4613 participants. Out of 6204, 93 current smokers were excluded (those who smoked at least one cigarette in last thirty days). Participants were also excluded due to missing data, (Sex = 239), (Age in years = 202), (Parents’ smoking status = 58 ), (Friends’ smoking status = 108), (Health education classes on harmful effects of smoking = 157), (Access to anti-smoking media = 82), (Exposure to billboard cigarette marketing = 208), (Exposure to newspaper/magazines cigarette marketing = 146), (Boys who smoke have more or less friends = 68), (Girls who smoke have more or less friends = 105), (Boys who smoke are more or less attractive = 116), (Girls who smoke are more or less attractive = 150), (People who smoke are more or less comfortable = 255), (Knowledge of harmful effects of smoking = 116), (Knowledge of harmful effects of second hand smoke = 64), (If your friends offered you a cigarette, would you smoke it = 113), (Do you think you will smoke a cigarette any time during next 12 months? = 50), (Do you think you will smoke a cigarette 5 years from now? = 70).

We found 11.6% (534) of nonsmoking students aged between 11 and 17 to be susceptible to smoking. Sex (*χ*^2^ = 0.021, *df* = 1, p value 0.866) and age (*χ*^2^ = 1.514, *df* = 1, p-value 0.218) were not significantly associated with susceptibility to smoking. Having parents (*χ*^2^ = 46.987, *df* = 2, p-value <0.001), or closest friend (*χ*^2^ = 130.0, *df* = 1, p-value <0.001) who smoke, were significantly positively associated with susceptibility. Perceptions that smokers are more comfortable at social gatherings (*χ*^2^ = 12.141, *df* = 1, p-value < 0.001); or smokers are more attractive than nonsmokers (*χ*^2^ = 19.354, *df* = 1, p-value < 0.001 for boys), (*χ*^2^ = 13.655, *df* = 1, p-value <0.001); having exposure to newspaper and magazines cigarette marketing (*χ*^2^ = 7.156, *df* = 1, p-value 0.007);were significantly positively associated with susceptibility to smoking. Students who had good knowledge about harmful effects of smoking (*χ*^2^ = 36.923, *df* = 1, p-value < 0.001); and had access to anti-smoking media (*χ*^2^ = 4.203, *df* = 1, p-value0.040); were significantly negatively associated with susceptibility to smoking. We did not find a significant association of susceptibility to smoke with: classes on harmful effects of smoking (*χ*^2^ = 1.964, *df* = 1, p-value 0.161), exposure to billboard cigarette marketing (*χ*^2^ = 3.502, *df* = 1, p-value 0.061), knowledge about harmful effects of second hand smoke (*χ*^2^ = 0.388, *df* = 1, p-value 0.533), perception that smoking boys have more friends (*χ*^2^ = 0.005, *df* = 1, p-value 0.946), and perception that smoking girls have more friends (*χ*^2^ = 0.686, *df* = 1, p-value 0.407) (Table 2).

**Table 2:** Baseline characteristics of non-smokers by smoking susceptibility status (n = 4613)

**Characteristics**	**Total**	**Non-susceptible n (%)**	**Susceptible n (%)**	*p-value
**Sex**				
Male	3028	2676 (88.4)	352 (11.6)	0.866
Female	1585	1403 (88.5)	182 (11.5)	
**Age in years (categorical)**				
Aged ≤ 13	1047	937 (89.5)	110 (10.5)	0.218
Aged > 13	3566	3142 (88.1)	424 (11.9)	
**Parents’ smoking status**				
None	2918	2651 (90.8)	267 (9.2)	<0.001
At least one parent smokes	1614	1363 (84.4)	251 (15.6)	
I don’t know	81	65 (80.2)	16 (19.8)	
**Friends’ smoking status**				
Donot have friends who smoke	3431	3142 (91.6)	289 (8.4)	<0.001
Have friends who smoke	1182	937 (79.3)	245 (20.7)	
**Classes on harmful effects of smoking**				
Did attend	2417	2122 (87.8)	295 (12.2)	0.161
Did not attend	2196	1957 (89.1)	239 (10.9)	
**Access to antismoking media**				
No	2434	2130 (87.5)	304 (12.5)	0.040
Yes	2179	1949 (89.4)	230 (10.6)	
**Exposure to billboards cigarette marketing**				
No	2908	2591 (89.1)	317 (10.9)	0.061
Yes	1705	1488 (87.3)	217 (12.7)	
**Exposure to newspapers/magazines cigarette marketing**				
No	3203	2859 (89.3)	344 (10.7)	0.007
Yes	1410	1220 (86.5)	190 (13.5)	
**Boys who smoke have more or less friends**				
Less friends or no difference	3597	3180 (88.4)	417 (11.6)	0.946
More friends	1016	899 (88.5)	117 (11.5)	
**Girls who smoke have more or less friends**				
Less friends or no difference	3892	3448 (88.6)	444 (11.4)	0.407
More friends	721	631 (87.5)	90 (12.5)	
**Boys who smoke are more or less attractive**				
Less attractive or no difference	4039	3603 (89.2)	436 (10.8)	<0.001
More attractive	574	476 982.9)	98 (17.1)	
**Girls who smoke are more or less attractive**				
Less attractive or no difference	4191	3729 (89.0)	462 (11.0)	<0.001
More attractive	422	350 (82.9)	72 (17.1)	
**People who smoke are more or less comfortable at social gatherings**				
Less comfortable or no difference	4281	3805 (88.9)	476 (11.1)	<0.001
More comfortable	332	274 (82.5)	58 (17.5)	
**Knowledge of harmful effects of smoking**				
Poor	635	516 (81.3)	119 (18.7)	<0.001
Good	3978	3563 (89.6)	415 (10.4)	
**Knowledge of harmful effects of secondhand smoke**				
Poor	4557	4028 (88.4)	529 (11.6)	0.533
Good	56	51 (91.1)	5 (8.9)	

*The p value has been calculated using Chi square test.

Univariate analysis indicates that nonsmoking students are more likely to be susceptible to smoking if they have: at least one parent who smokes (OR = 1.82; 95% CI [1.52-2.19]); closest friends who smoke (OR = 2.84, 95% CI [2.36, 3.42]), and exposure to newspaper/magazines cigarette marketing(OR = 1.29, 95% CI [1.07, 1.56]). Students having perceptions that smoking boys are more attractive than nonsmokers (OR = 1.70, 95% CI [1.34, 2.16]); smoking girls are more attractive than nonsmokers (OR = 1.66, 95% CI [1.26-2.17]); and smokers are more comfortable at social gatherings (OR = 1.69, 95% CI [1.25-2.28]) were also more likely to be susceptible to smoke. Students who had access to anti-smoking media(OR = 0.82, 95% CI [0.68-0.99]), and knowledge of harmful effects of smoking(OR = 0.50, 95% CI [0.40-0.63]) were less likely to be susceptible to smoking. The students’ susceptibility to smoking was not significantly associated with age, sex, classes on harmful effects of smoking, exposure to billboard cigarette advertisements, perceptions that smokers have more friends, and knowledge about harmful effects of second hand smoke (Table 3).

**Table 3:** Factors associated with smoking susceptibility among current non-smokers (n = 4613)

	**Univariate analysis**		**Multivariate analysis**	
**Characteristics**	**OR**^**a **^**(95% CI)**	**p-value**	**OR**^**b **^**(95% CI)**	**p-value**
**Sex**				
Male	1		1	
Female	0.98 (0.81-1.19)	0.886	1.53 (1.24-1.89)	<0.001
**Age in years (categorical)**				
≤ 13	1		1	
> 13	1.14 (0.92-1.43)	0.219	1.06 (0.84-1.33)	0.597
**Parents’ smoking status**				
None	1		1	
At least one parent smokes	1.82 (1.52-2.19)	<0.001	1.64 (1.35-1.99)	<0.001
I don’t know	2.44 (1.39-4.28)	<0.001	1.51 (0.82-2.76)	0.179
**Friends’ smoking status**				
do not have friends who smoke	1		1	
Have friends who smoke	2.84 (2.36-3.42)	<0.001	2.77 (2.27-3.40)	<0.001
**Classes on harmful effects of smoking**				
Did attend	1		1	
Did not attend	1.13 (0.95-1.36)	0.161	1.16 (0.95-1.40)	0.126
**Access to antismoking media**				
No	1		1	
Yes	0.82 (0.68-0.99)	0.041	0.73 (0.59-0.89)	0.002
**Exposure to billboards cigarette marketing**				
No	1		1	
Yes	1.19 (0.99-1.43)	0.062	0.98 (0.78-1.22)	0.863
**Exposure to newspapers/magazines cigarette marketing**				
No	1		1	
Yes	1.29 (1.07-1.56)	0.008	1.30 (1.03-1.65)	0.026
**Boys who smoke have more or less friends**				
Less friends or no difference	1		1	
More friends	0.99 (0.79-1.23)	0.946	0.78 (0.58-1.04)	0.090
**Girls who smoke have more or less friends**				
Less friends or no difference	1		1	
More friends	1.10 (0.87-1.41)	0.408	0.96 (0.70-1.34)	0.851
**Boys who smoke are more or less attractive**				
Less attractive or no difference	1		1	
More attractive	1.70 (1.34-2.16)	<0.001	1.36 (0.99-1.85)	0.051
**Girls who smoke are more or less attractive**				
Less attractive or no difference	1		1	
More attractive	1.66 (1.26-2.17)	<0.001	1.30 (0.91-1.85)	0.141
**People who smoke are more or less comfortable**				
Less comfortable or no difference	1		1	
More comfortable	1.69 (1.25-2.28)	0.001	1.32 (0.94-1.85)	0.101
**Knowledge of harmful effects of smoking**				
Poor	1		1	
Good	0.50 (0.40-0.63)	<0.001	0.54 (0.43-0.69)	<0.001
**Knowledge of harmful effects of secondhand smoke**				
Poor	1		1	
Good	0.74 (0.29-1.87)	0.535	0.62 (0.24-1.62)	0.335

OR^a^ = unadjusted odds ratio.

OR^b^ = odds ratios adjusted for gender, age, parents smoking status, friend smoking status, education of harmful effects of smoking, access to antismoking media, exposure to billboards cigarette marketing, exposure to newspaper/magazines cig marketing, expected social outcomes of smoking, knowledge of harmful effects of smoking, knowledge of harmful effects of secondhand smoke.

CI = confidence intervals.

Further analysis using multiple logistic regressions also provided consistent results. Additionally, students who were females (OR = 1.53, 95% CI [1.24-1.89]) were more likely to be susceptible to smoking as compared to males. Students who had at least one smoking parent (OR = 1.64, 95% CI [1.35-1.99]); smoking friends (OR = 2.77, 95% CI [2.27-3.40]); and exposure to cigarette advertisements in newspapers and magazines (OR = 1.30, 95% CI [1.03-1.65]; were more likely to be susceptible to smoking. However, students who had access to antismoking media (OR = 0.73, 95% CI [0.59-0.89], and had knowledge of harmful effects of smoking (OR = 0.54, 95% CI [0.43-0.69] were less likely to be susceptible to smoking after adjustment for all of the independent variables (Table 3).

Furthermore, we stratified the analysis based on ever smoking and never smoking categories. We found that students who were ever smokers and were 13 years or older (OR = 1.99, 95% CI [1.22-3.24]); and had a smoking friend (OR = 2.75, 95% CI [1.82-4.14]) were more likely to be susceptible to smoking. Moreover students who were never smokers and were females (OR = 1.80, 95% CI [1.43-2.28]); had a smoking parent (OR = 1.65, 95% CI [1.31-2.07]); smoking friends (OR = 2.12, 95% CI [1.66-2.71]), and had exposure to cigarette marketing (OR = 1.40, 95% CI [1.10-1.78]) were more likely to be susceptible to smoking. Never smokers who had knowledge of harmful effects of smoking (OR = 0.48, 95% CI [0.37-0.62]); or had access to antismoking media (OR = 0.72, 95% CI [0.57-0.92]) were less likely to be susceptible to smoking (Table 4).

**Table 4:** Multivariate analysis of ever smoked and never smoked current non-smokers with smoking susceptibility (n = 4613)

	**Ever smoked (n = 529)**		**Never smoked (n = 4084)**	
**Characteristics**	**OR**^**b **^**(95% CI)**	**p-value**	**OR**^**b **^**(95% CI)**	**p-value**
**Sex**				
Male	1		1	
Female	1.02 (0.57-1.82)	0.930	1.80 (1.43-2.28)	<0.001
**Age in years (categorical)**				
≤ 13	1		1	
>13	1.99 (1.22-3.24)	0.006	0.92 (0.70-1.19)	0.537
**Parents’ smoking status**				
None	1		1	
At least one parent smokes	1.23 (0.83-1.83)	0.287	1.65 (1.31-2.07)	<0.001
I don’t know	1.65 (0.43-6.28)	0.463	1.62 (0.82-3.23)	0.167
**Friends’ smoking status**				
Do not have friends who smoke	1		1	
Have friends who smoke	2.75 (1.82-4.14)	<0.001	2.12 (1.66-2.71)	<0.001
**Knowledge of harmful effects of smoking**				
Poor	1		1	
Good	0.69 (0.40-1.19)	0.189	0.48 (0.37-0.62)	<0.001
**Access to anti-smoking media**				
No	1		1	
Yes	1.12 (0.73-1.70)	0.598	0.72 (0.57-0.92)	0.007
**Exposure to newspaper/magazines cigarette marketing**				
No	1		1	
Yes	1.30 (0.88-2.15)	0.156	1.40 (1.10-1.78)	0.006

OR^b^ = Odds ratios adjusted for gender, age, parents’ smoking status, friends’ smoking status, Knowledge of harmful effects of smoking, access to antismoking media, exposure to cigarette marketing.

## Discussion

Adolescents with smoking parents and friends; who had exposure to newspapers and magazines cigarette advertisements; were females; and those who perceived that smoking yields positive social outcomes; were more likely to experiment with cigarette smoking. Students who had good knowledge about harmful effects of smoking; and had access to antismoking media were less likely to experiment with cigarette smoking.

This study found that one in ten of all adolescents was susceptible to initiate smoking. The prevalence is higher than Afghanistan (8.8%) and Iran (8.7%); however it is lower than many countries in the region, like India (15.5%), Bangladesh (13.2%), and Nepal (16.4%) [15]. According to the WHO, smoking is a rising epidemic in developing countries , given the validation of susceptibility to smoke as the predictor of cigarette experimentation, this finding in Pakistan should serve as a warning sign, telling us about the new smokers which might enter in the pool in near future.

The finding about females being more prone to cigarette experimentation is important to understand evolving gender role associated with smoking initiation among adolescents. WHO reported that prevalence of smoking among females is on the rise and females are being targeted in the marketing strategies of cigarette industry [16]. GYTS Lahore 2008 (7% as compared to 6.7% in females and males respectively), Yemen (27.4% as compared to 22.1%), Indonesia (Medan) (98.9% as compared to 97.7%) , Nepal (Biratnagar) (4.0% as compared to 2.8% ), Canada (51.6% as compared to 48.4% ), Bangui 2008 (18.3% as compared to 16.1%); have also found a higher susceptibility among females, which could be suggestive of rising susceptibility among females [15]. We also found that more female students (71.5%) as compared to males (64.7%) had observed cigarette promotions and advertisements in newspapers and magazines. One reason of increased susceptibility of females to smoke could be the result of the tobacco industry’s marketing target. However, there are more evolving social and behavioral aspects to be understood. Mejia, Raul, et al. found that girls with egalitarian gender role attitudes had higher odds of smoking [17]. Furthermore, Michael G., et al. also found that prevalence of susceptibility to smoke among urban female students (3.5%) was higher than rural female students (1.7%) [18]. It is usually assumed that urban females have an egalitarian gender role attitude, and this might be true for the school going girls of the three cities selected in this study. It could be due to rapidly changing role of gender in an evolving society like Pakistan.

In this regard, our findings of social outcomes expected out of smoking are also important. Students who expected positive social outcomes out of smoking have higher susceptibility to smoke. Anna V., et al. found positive social outcomes expected out of smoking to be the strongest predictor of susceptibility to smoke [19]. Limited research is available to explore young Pakistanis’ perspective. Our finding of females being more susceptible to smoke, and importance of expected social outcomes of smoking; support the call to move away from gender blind tobacco control policies. Furthermore, it highlights the need of future research in Pakistani socio-cultural context. We need to find out the difference of smoking susceptibility prevalence between rural and urban setups of Pakistan. Additionally qualitative research is needed to understand the evolving norms of Pakistani society.

In general, there is a strong association between parents’ and peers’ smoking status; and smoking initiation among adolescents [20,21]. Our findings are consistent with other findings from the country [22]; we found friend’s smoking status to be the strongest predictor of susceptibility to smoking followed by parents’ smoking status. It is argued by Wilkinson, Anna V., et al. that having close friends who smoke may not definitely mean that it was the friends who caused the participant to smoke. It could be due to the fact that people tend to choose friends based on shared characteristics [19,23]. We could not assess this issue in our study. The exact nature of peer social context in determining Pakistani youth’s susceptibility to smoke needs to be explored. And anti-tobacco policies need to focus on the family context during the development primary prevention strategies targeting the Pakistani youth.

The importance of health education at schools, as an intervention against tobacco use has been found to be an important protective factor against smoking. We found that having good knowledge about harmful effects of smoking; and access to anti-smoking media; served as protective factors against susceptibility to smoke. Interestingly, we did not find a significant association of educational classes on harmful effects of smoking with susceptibility to smoke. These two findings are suggestive of ineffectiveness of the health educational classes that were conducted. Although Lindberg LC observed that knowledge based interventions at school level alone do not impact behavior [24], but we do not know about the extent or nature of strategies used in classes on harmful effects of smoking. School based interventions need to be improved further to avail their maximum benefit as a protective factor. Nevertheless, the importance of education regarding primary prevention cannot be undermined and our findings regarding knowledge about harmful effects of smoking and access to anti-smoking media underscore its importance.

Susceptibility has been used as a predictor for experimentation with cigarette smoking. We further went to assess the group of individuals who have already experimented with smoking and found that those who were 13 years old or older; and had a close friend who smoked were at a higher risk of attempting cigarette smoking again in future. For the first timers (those who have never experimented with cigarette smoking), however, it was observed that many more factors were involved along with age factor, and peer smoking status. They were at a higher risk of experimenting if their parents smoked; and if they were exposed to the tobacco marketing tactics. Counteracting the forces that might pull these young adults towards smoking, it is imperative to focus on increasing their knowledge of harmful effects of smoking through various means including effective health education classes teaching resistance skills; and increasing their knowledge of smoking related health hazards.

This study is the first one to report association of various risk factors with susceptibility to smoke from Pakistan. Further research will be needed to confirm these findings and determine whether these findings can be generalized to our national population. Our study has various limitations. Firstly, data from GYTS are self-reported, although Brener et al. have found that the results generated from self-reported data are reliable [25]. Secondly GYTS was administered only to the youth who are enrolled in school, and were present on the day of survey. We did not have any information about the Pakistani youth who do not attend school. This may have not have direct effect on our findings, because young adolescents who do not attend school are mostly from low socio-economic groups who cannot afford to pay for schooling. That particular sub-group of population is likely to have entirely different characteristics and distribution of health risk behaviors including susceptibility to smoking and determinants associated with it. Therefore the findings of this study may not be generalizable to that socio-economic group of population. Moreover, we could not deal with the issue of non-responders, as this survey was conducted years earlier and the present study was only based on secondary analysis of existing data. Lastly, this survey was conducted in only three cities of Pakistan; therefore the findings may be conservative. However, these cities were a mix of urban and rural populations, so our findings may still be generalizable to fairly larger chunk of Pakistani adolescents.

## Conclusion

Pakistani youth having smoking parents and friends, who are females, or have exposure to newspaper/magazines cigarette marketing, are more susceptible to initiate smoking. While knowledge of harmful effects of smoking and access to anti-smoking media served as protective factors against susceptibility to smoking. Preventive efforts need to focus on various social and behavioral aspects to make the prevention programs gender and culture sensitive; with more emphasis on urban female youth, smoking adults who are parents to young children, and improved school based interventions like classes on harmful effects of smoking.

## Competing interest

The authors declare that they have no competing interests.

## Authors’ contribution

KS conceived the idea and all authors designed the study; SZ and SR carried out statistical analyses; all authors contributed to interpreting the results; SK drafted the manuscript; KS supervised the study, all authors saw and approved the final manuscript.
